# Impact of dynamic bond strength on the training of liquid crystal elastomers

**DOI:** 10.1039/d5sc07491f

**Published:** 2025-12-04

**Authors:** E. Ghimire, I. S. Appen, C. A. Lindberg, L. Blagitz de Abreu e Silva, S. J. Rowan

**Affiliations:** a Pritzker School of Molecular Engineering, University of Chicago Chicago Illinois 60637 USA stuartrowan@uchicago.edu; b University of Chicago Laboratory Schools Chicago Illinois 60637 USA; c Department of Chemistry, University of Chicago Chicago Illinois 60637 USA; d Center for Molecular Engineering, Argonne National Laboratory 9700 S. Cass Ave. Lemont IL 60434 USA

## Abstract

Soft materials can be considered trainable when their functional/mechanical properties can be systematcially improved upon exposure to repeated external environmental stresses, such as mechanical loads. One class of material that has the potential to be trainable is dynamic liquid crystal elastomers (dLCEs), which are lightly crosslinked polymer networks that contain both anisotropic liquid crystalline molecules (mesogens) and dynamic bonds. In this work, the trainability of dLCEs is studied by utilizing the mechanical adaptability of mesogenic units in combination with reprogrammability and reconfigurability enabled by the dynamic bonds. The effects of dynamic bond strength on the thermomechanical and liquid crystalline properties of LCEs were studied by synthesizing a series of aza-Michael based dynamic LCEs by incorporating diamine crosslinkers containing either no dynamic bonds, disulfide bonds, or diselenide bonds. Compared to the two other systems, the LCE containing diselenide bonds exhibited significantly higher toughness at room temperature. The effects of mechanical strain on the trainability of the films was studied under slightly elevated temperatures, where diselenide-containing dLCEs exhibited enhanced actuation and stiffer mechanical properties with higher strain levels. Moreover, using the combined thermal and mechanical training protocols, spiral actuators fabricated from the dLCEs demonstrated self-sustained motion upon heating them above their nematic to isotropic transition temperatures. In addition, retrainability was demonstrated in diselenide LCEs by generating two distinct actuator shapes from a single sample, where blue light was used to spatially control the effective regions of training. Overall, this study examines how the strength of dynamic bonds influences the properties and trainability of LCEs and how functional responses can be tailored by applying different training protocols.

## Abstract 2

Liquid crystal elastomers that contain bonds of varying strengths (C–C, S–S, Se–Se) were prepared. The weaker, more dynamic Se–Se-based materials showed enhanced toughness and exhibited trainable behavior that allowed access to tunable properties.

## Graphical abstract

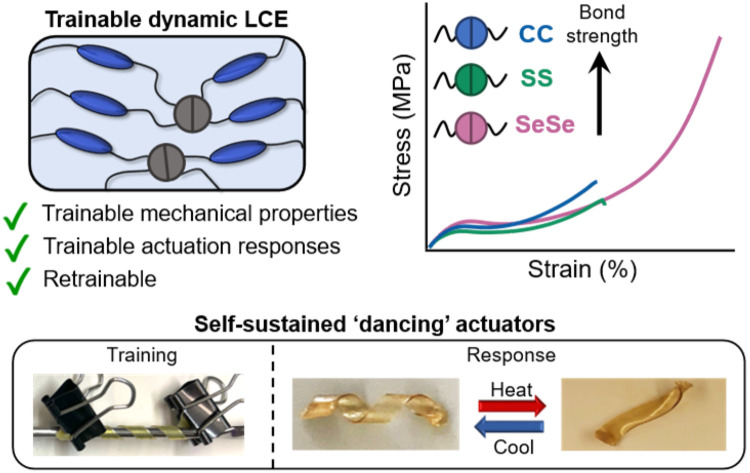

## Introduction

**Fig. 1:**
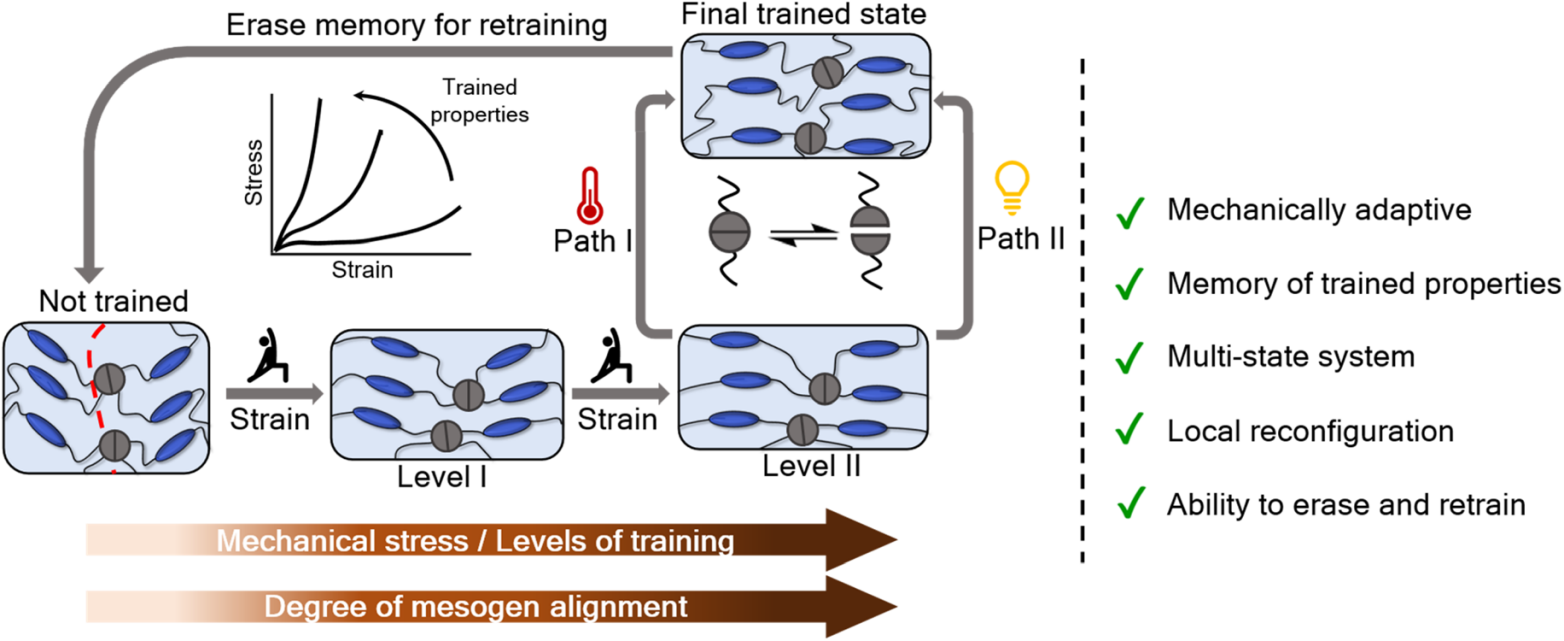
Schematic showing the concept of training dynamic liquid crystal elastomers.

Biological systems, like muscles and bones, naturally evolve by continuously reconfiguring themselves to adapt to varying environmental stresses.^1,2^ In contrast, most synthetic materials lack this ability for self-reconstruction and so when repeatedly exposed to environmental factors and mechanical stress, they tend to degrade over time leading to material damage and a decline in functionality. Inspired by these biological systems, researchers have recently begun exploring mechanically trainable materials,^3–6^ in which the materials are able to utilize the external stress of their environment in favorable ways such as the initiation of mechano–chemical reactions for strength enhancement^4,5^ and reorganization of macroscopic networks for tunable elasticity.^6^ Although the intrinsic mechanism of material training varies by system, generally, the stimuli applied to the material are directed to specific constituents, the response of which leads to property enhancement.^7^ In addition, unlike the traditional approach of material design, where the desired properties are achieved by tuning the composition or formulation during synthesis, trainable materials can be exposed to repeated and/or different training protocols to achieve a range of properties post-synthesis.^7^ Training protocols for mechanically trainable materials involve instructing a material system to adapt under stress by controlling the type, intensity, and duration of applied stimuli to achieve desired responses. Some specific examples of trainable materials include mechanoresponsive self-growing hydrogels,^5^ self-strengthening organogels,^4^ and thermo-mechanically responsive dual network hydrogels.^8^ For example, Matsuda *et al.* synthesized double network hydrogels that self-strengthen under mechanical stress, mimicking muscle training through repeated structural breakdown and reconstruction of the network.^5^ Similarly, Esser-Kahn and coworkers synthesized materials that, in the presence of piezoelectric particles, harness mechanical vibrations to induce crosslinking thereby progressively enhancing the system's modulus in response to a controlled external stimulus.^4^ These approaches exploited mechano-responsive polymerizations in which the training process generated reactive species that subsequently induced irreversible polymerization of monomeric species present in the system. Another type of training approach has been to confine disordered macroscopic networks of polymeric foams in compressive environments to achieve metamaterials with different Poisson's ratios.^6^ While material training has enabled many desirable properties, most of the trainable systems are irreversible in nature, therefore, they are not retrainable. Recently, Gowen *et al.* demonstrated retrainability in liquid crystal elastomer (LCE) based metamaterials by training one type of function (auxetic behavior), erasing it and retraining for a different type of function (allostery), by leveraging the strain-induced mechanical adaptability and the thermal shape memory recovery of LCEs.^9^ Building on this concept, it is desirable to explore the training of LCEs in more accessible geometries, such as films, and understand how training can be used to alter functional properties such as mechanical response and actuation. To this end, this work investigates the trainability of dynamic liquid crystal elastomers (dLCEs), where the multi-state mechanical adaptability of LCEs is combined with the reprogrammability and reconfigurability enabled by the dynamic bonds (Fig. 1).

LCEs are a class of stimuli-responsive material characterized by their unique optical and thermomechanical properties.^10–14^ They are lightly crosslinked polymer networks containing covalently bonded liquid crystal units (mesogens), resulting in materials that combine the entropic elasticity of rubber with the anisotropic properties of small-molecule liquid crystals.^14^ Unless the mesogens are aligned into a monodomain during the synthesis, the LCE will be in a polydomain state at equilibrium, where micron/nano sized domains have locally aligned mesogens and the global domain orientation is random.^15,16^ One of the distinctive features of LCEs is their “soft elasticity.” This property allows the material to undergo significant deformation without experiencing a substantial increase in stress within a specific strain range. This phenomenon is attributed to the strain-induced rotation of mesogens, which leads to the formation of a stress plateau under uniaxial tension.^14,17^ Beyond the soft elastic plateau, the majority of the mesogens are aligned in the direction of strain leading to anisotropy in the network.^18,19^ Although LCEs have been extensively studied for applications across diverse fields such as soft robotics,^20–23^ impact mitigation,^24,25^ biomedical devices,^26–28^ adhesives,^29–31^ and optical information storage,^32–34^ their potential as trainable systems has not been fully explored. LCEs possess the capacity to rearrange their mesogenic units in response to mechanical deformation, making them suitable as mechanically trainable materials. Incorporating dynamic bonds into LCEs further enables the mesogen alignment to be “locked in” during training, enabling access to additional functional properties such as actuation.^35–38^

Dynamic bonds are reversible bonds that can break and reform usually upon exposure to stimuli such as heat, light, pH, *etc.*, ideally without any side reactions.^39^ Dynamic bonds have been incorporated into materials to access different functionalities including reprocessability, shape memory, pluripotency, and self-healing properties.^40–44^ Incorporating dynamic bonds in LCEs offers further advantages as they enable the ability to realign the mesogens, through a combination of an applied strain and dynamic bond-induced stress relaxation of the network, post synthesis.^36–38^ By performing dynamic bond exchange while orienting mesogen alignment in different directions, a range of actuation capabilities can be achieved from the same LCE film.^37^ Therefore, incorporating dynamic chemistries has been of growing interest in the LCE community, particularly to achieve complex patterns of mesogen alignment.^36–38^ A range of dynamic LCEs have been synthesized that include a variety of dynamic covalent chemistries such as transesterification,^35^ boronic transesterification,^45^ disulfide exchange,^46^ and diselenide exchange.^47^ As training is conducted under mechanical stress, it is important to understand how different dynamic bonds influence the mechanical behavior, such as toughness, of the LCEs as this can better inform their ability to dissipate energy during training.

Reported herein is an investigation of dLCEs as mechanically trainable materials where the alignment of mesogens along with dynamic bond exchange was utilized to impart trainability to the material. The aza-Michael reaction was chosen to synthesize dLCEs as it allows a straightforward and efficient way to synthesize the networks.^48^ In addition, there are a wide variety of diamines available that can be used as crosslinkers, allowing facile access to a range of LCEs. Specially, cystamine (2,2′-dithiobisethanamine) and selenocystamine (2,2′-diselenobisethanamine) are commercially available or easily accessible and allow access to dynamic LCEs whose exchange kinetics can be varied by simply altering the type of diamine crosslinker. This strategy also helps maintain comparable dynamic bond concentrations across samples while reducing variations in the overall network structure. Disulfide and diselenide bonds are chalcogen based dynamic bonds that are multiresponsive making them interesting candidates for accessing training materials.^49–52^ Both disulfides and diselenides undergo similar mechanisms of dynamic exchange where they can dissociate to form radicals (under heat or light) that can then further exchange with the remaining dichalcogenides through an associative process.^53–56^ Comparatively, the bond energy of disulfides (240 kJ mol^−1^) is higher than that of diselenides (172 kJ mol^−1^), hence disulfides are active under stimuli with relatively higher energies than that of diselenides.^57^ For example, both disulfides and diselenides are active under light, however, disulfides require light in the UV region, while diselenides can be activated with visible light.^54^ While disulfide and diselenide bonds have been incorporated into LCEs previously,^46,47,58^ there is a lack of systematic study exploring how the difference in bond energies between these systems impact the properties of LCEs, such as thermal transitions, material toughness, stress relaxation, and mesogen alignment under strain.

## Results and discussion

**Fig. 2:**
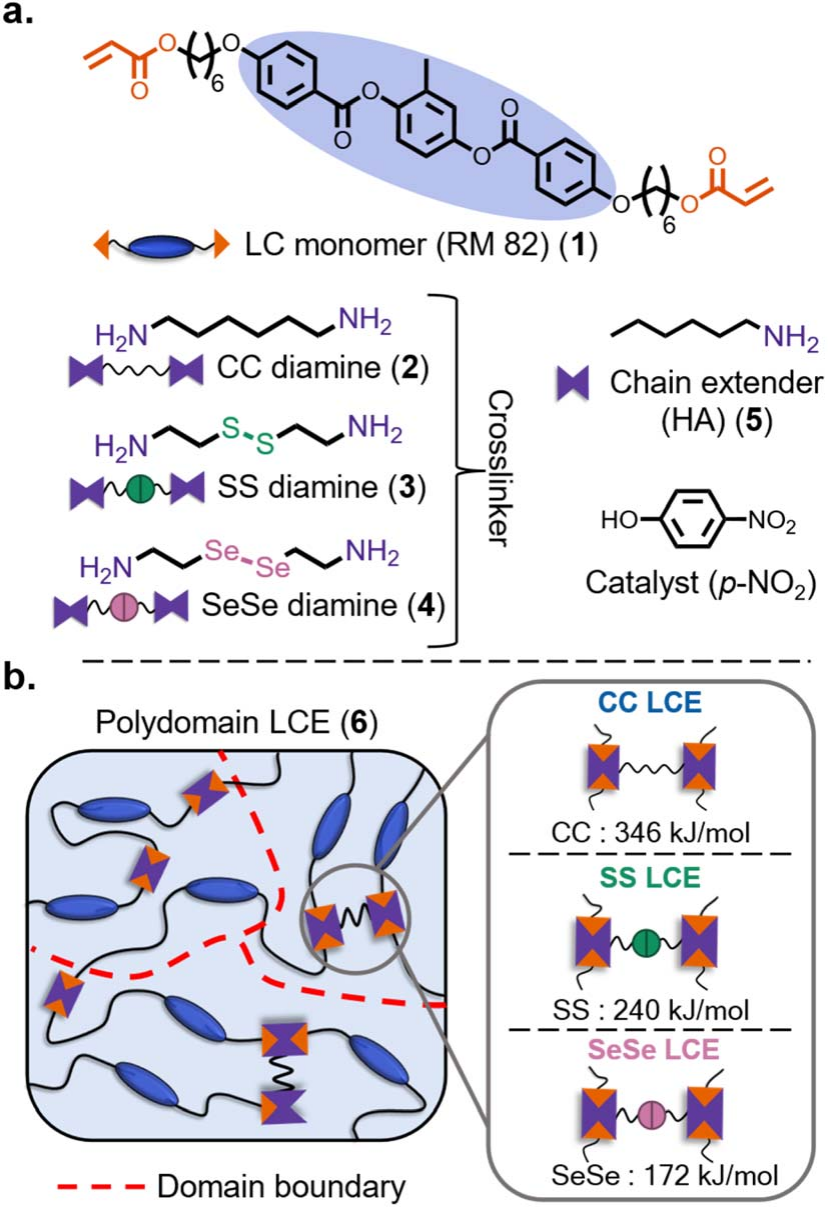
(a) Chemical structures of the LCE network components (b) a schematic illustration of the polydomain LCE, highlighting the crosslinking moieties that distinguish the three LCE systems: 6CC, 6SS, and 6SeSe, based on the type of crosslinker used, along with the bond strengths of the three different bonds within the crosslinkers. Red dashed lines labelled as ‘domain boundary’ separate regions of locally aligned mesogens within the polydomain samples.

The initial work focused on exploring how dynamic bond strength influences the thermomechanical and liquid crystalline properties of this class of LCEs. To investigate this, a commercially available liquid crystalline diacrylate monomer (RM 82) (1), three different diamine crosslinkers (2, 3, or 4), and a monoamine chain extender (5) were reacted using aza-Michael chemistry to obtain a control nondynamic polydomain LCE (6CC) and two polydomain dynamic LCEs (6SS and 6SeSe) (Fig. 2a and b). Using this approach allows access to LCEs whose major difference is in their crosslinking moieties that contain bonds of different bond strengths, CC (346 kJ mol^−1^), SS (240 kJ mol^−1^), and SeSe (172 kJ mol^−1^),^57^ depending on the choice of crosslinker (2, 3, or 4, respectively). The reactions were carried out at 60 °C in presence of *p*-nitrophenol (*p*-NO_2_) as the catalyst, which has previously been shown to enhance the reactivity of aza-Michael reactions.^48^ Each LCE system, 6CC, 6SS, and 6SeSe was synthesized with three target molecular weights between crosslinks (*M*_c_) of 2 kg mol^−1^, 3.5 kg mol^−1^, and 5 kg mol^−1^, thus forming 6CC-*x*, 6SS-*x*, and 6SeSe-*x*, where *x* is the target *M*_c_ in kg mol^−1^. To achieve the target *M*_c_, the ratios of the crosslinker to the chain extender was varied as shown in Table S1. After gelation, the networks were thoroughly washed with acetone using a Soxhlet extractor apparatus to remove the sol fraction and catalyst. The washed gels were then dried under vacuum at 60 °C overnight, after which they were melt processed to obtain uniform polydomain films of the LCEs with a thickness around 0.5 mm.^38^ Thermogravimetric analysis confirmed solvent removal and revealed that all samples exhibited primary thermal degradation onset above 230 °C, which is at least 50 °C above any temperature used for the subsequent material characterizations (Fig. S1). Infrared spectroscopy measurements confirmed similar reactivity across all three samples (Fig. S2).

**Fig. 3:**
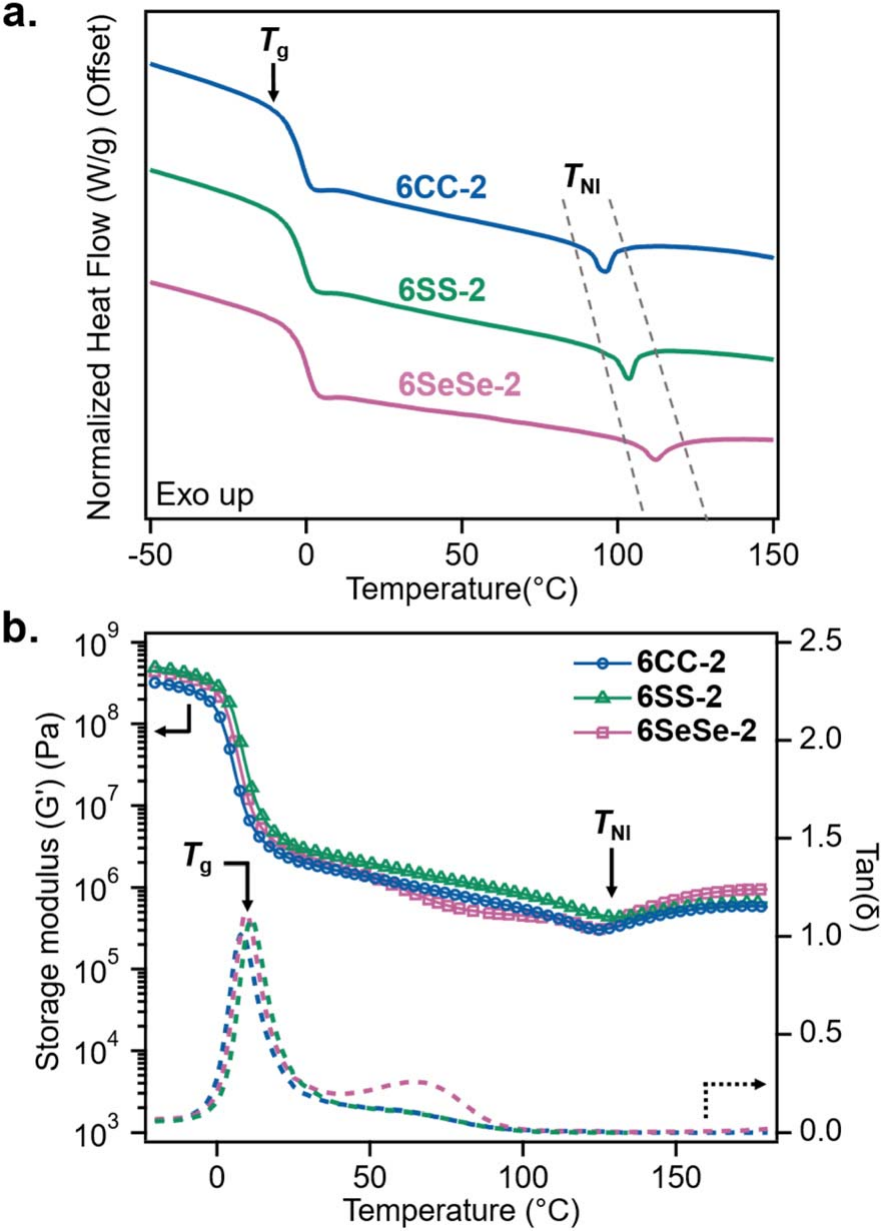
Thermomechanical data for 6CC-2, 6SS-2, and 6SeSe-2 (a) differential scanning calorimetry thermograms showing the glass transition temperatures (*T*_g_) and the nematic to isotropic transition temperatures (*T*_NI_) (temperature ramp rate = 10 °C min^−1^) (b) shear rheology dynamic temperature sweeps (temperature ramp rate = 3 °C min^−1^, frequency = 1 Hz, parallel plate geometry) showing storage moduli (solid lines with markers) and tan delta (dashed lines) of the LCEs.

The thermal transition temperatures of the LCEs were measured using differential scanning calorimetry (DSC). Within each LCE system, 6CC, 6SS, and 6SeSe, the glass transition temperature (*T*_g_) as well as the nematic to isotropic transition temperature (*T*_NI_) decreased with an increase in *M*_c_ (Table S2 and Fig. S3–S5). Fig. 3a compares the effects of crosslinkers 2, 3, and 4 on the thermal properties of 6CC-2, 6SS-2, and 6SeSe-2, respectively. The *T*_g_'s of all three systems were very similar (between 2 °C and −2 °C), however the *T*_NI_'s slightly increased with a decrease in the bond energy of the dynamic bond present in the crosslinker. The *T*_NI_'s, measured as the midpoint of the endothermic peaks in the DSC thermograms, were 97 °C, 100 °C, and 110 °C for 6CC-2, 6SS-2, and 6SeSe-2 respectively, which suggests that the packing stability of the mesogens moderately increases with a decrease in the bond strength in the crosslinking unit. This behavior may arise because crosslinkers with weaker bonds can thermally dissociate more readily, enabling improved network rearrangement and the formation of more stable mesogenic domains. In addition, the thermomechanical behavior of the networks was studied by conducting dynamic temperature ramp measurements using shear rheology. The *G*′ curves in Fig. 3b illustrate that 6CC-2, 6SS-2, and 6SeSe-2 display comparable storage moduli across the entire temperature range probed (−20–180 °C). The first peak in the tan *δ*′s of the three LCEs overlap indicating similar *T*_g_'s, which is in accordance with the DSC results. The temporary drop in storage moduli at *ca.* 125 °C is indicative of the transition of LCEs from the nematic to isotropic state, which is observed to be present across all three systems. It is interesting to note while a slight increase in *T*_NI_ was observed in the DSC measurements, the *T*_NI_ values of the LCEs were similar when measured in terms of viscoelastic response even though the bond energy of the dynamic bond in the crosslinker changes. A broader transition is typically observed in dynamic temperature ramp experiments due to the presence of paranematic behavior above the *T*_NI_.^59^ Therefore, the small difference in *T*_NI_ detected in the DSC measurements could have been masked in the dynamic temperature ramp results. Typical to LCEs, all three samples exhibit a second peak/shoulder in tan *δ* after the first peak (*T*_g_), which results from the internal relaxation of nematic director under the oscillatory shear measurements.^60^ However, it is noteworthy that the 6SeSe-2 has a more distinct secondary tan *δ* peak relative to the other two samples suggesting higher dissipative properties in this material.^59^ In addition, all three samples show a storage modulus higher than the loss modulus across the frequency range of 0.1–100 Hz, indicating a solid-like response. Notably, the convergence of the storage and loss moduli occurs at lower frequencies for samples containing weaker bonds in the crosslinker, suggesting that dynamic bonds contribute to energy dissipation in LCEs at lower frequencies (Fig. S6–S8).

**Fig. 4:**
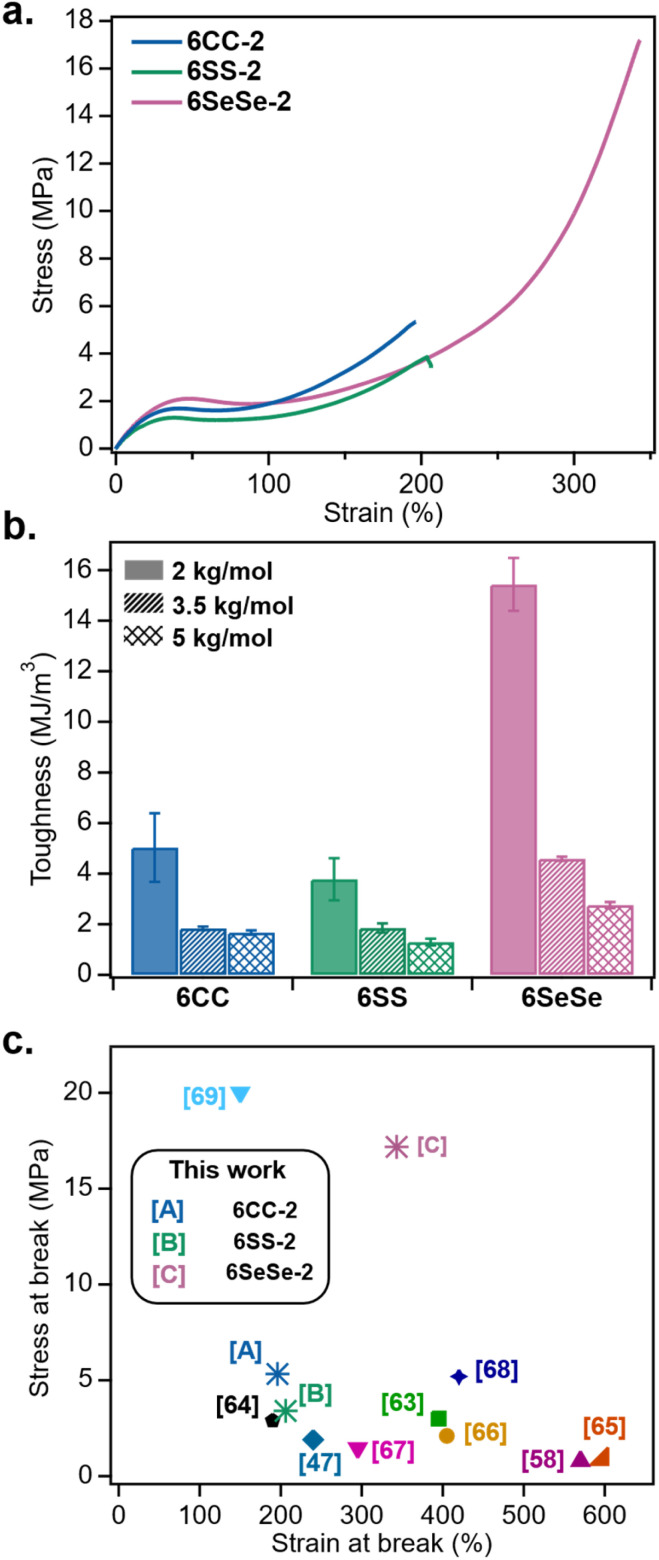
Room temperature mechanical properties of LCEs (a) representative uniaxial tensile curves of 6CC-2, 6SS-2, and 6SeSe-2 (strain rate = 1% per sec), (b) comparison of toughness (area under the stress–strain curve) of the LCE films with different bond strengths and *M*_c_, (c) stress and strain-at-break for 6CC-2, 6SS-2, 6SeSe-2 compared to dynamic LCEs reported in the literature-siloxane,^63^ allyl sulfide,^64–66^ disulfide,^58,67^ diselenide,^47,69^ and thiourea.^68^

To further explore the impact of dynamic bond strength in these LCEs, room temperature stress–strain properties were evaluated. Fig. 4a shows the representative tensile curves for 6CC-2, 6SS-2 and 6SeSe-2. All three samples exhibit three mechanical regions that are characteristic of polydomain LCEs: an elastic regime at low strains, followed by a soft elastic plateau and then a strain hardening regime at larger strains.^14^ While the Young's moduli for the three samples were similar (between 11 and 14 MPa), the major difference observed in the stress–strain behavior was the ultimate failure strain and stress-at-break, which were much higher for 6SeSe-2 compared to 6CC-2 and 6SS-2 (Fig. 4a). In addition, the range of the soft elastic plateau, determined based on the intersecting points of three tangent lines drawn on the tensile curves (Fig S9a),^14^ increased as the bond strength decreased, indicating less resistance from the network during mesogen rotation in the presence of weaker bonds (Fig. S9b). To determine the effect of bond strength in toughness, the area under the stress–strain curves were integrated and plotted (Fig. S10–S12 and 4b). Within each crosslinker type (6CC, 6SS, and 6SeSe), samples with lower crosslinking densities were found to exhibit lower toughness, a behavior previously reported in LCEs.^24,61,62^ At each crosslinking density, the toughness values of 6CC and 6SS were comparable within experimental error, however, the 6SeSe films consistently exhibited a significantly higher toughness. Notably, the average toughness of 6SeSe-2 was 15.44 MJ m^−3^, compared to 5.03 MJ m^−3^ for 6CC-2 and 3.78 MJ m^−3^ for 6SS-2, representing an increase of approximately 3–4 times. Similarly, the less crosslinked 6SeSe-3.5 exhibited a roughly 2.5 times increase in toughness compared to that of 6CC-3.5 and 6SS-3.5 and the toughness for 6SeSe-5 was 1.5–2 times higher than that of 6CC-5 and 6SS-5. The decrease in toughness difference upon increase in *M*_c_ suggests that the concentration of the dynamic bond (SS, and SeSe), in the film contributes significantly to the resulting mechanical properties. 6SeSe-2 when compared to some of the other dynamic LCEs reported in the literature showed significantly better mechanical properties (Fig. 3c).^47,58,63–69^ The stress-at-break for eight of the nine literature data presented in Fig. 4c fall below 5 MPa. Ref. 69, which is also an LCE system containing SeSe bonds, was reported with a high stress-at-break of around 20 MPa, due to additional hydrogen bonding, which also explains why it exhibits a relatively low extensibility of around 150%.^69^6SeSe-2 has a stress-at-break of *ca.* 17 MPa with an ultimate strain above 340%, resulting in its relatively high toughness value (15.44 MJ m^−3^). Diselenide bonds are known to act as mechanophores at room temperature, breaking under mechanical stress to generate radicals.^70^ We believe that the enhanced toughness observed in these diselenide-containing LCEs results from a combination of stress dissipation as a result of diselenide bond scission and mesogen reorientation along the strain direction. It is worthy of note that the incorporation of other energy dissipating moieties such as slidable crosslinks has also been previously shown to enhance the toughness in LCEs.^71^ Typical for polydomain LCEs, all three films (6CC-2, 6SS-2, and 6SeSe-2) transitioned from an opaque to a more transparent state upon stretching, which is consistent with the transformation from a polydomain to a monodomain state (Fig. S13).^13^

**Fig. 5:**
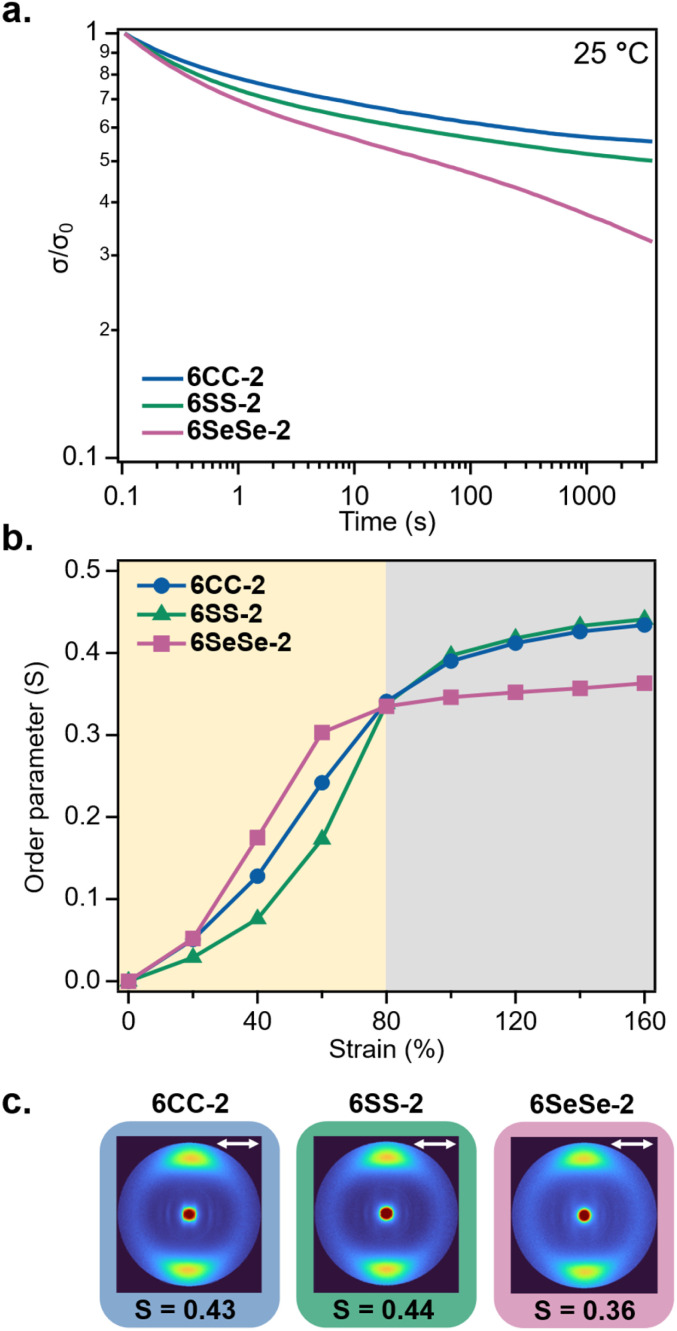
Dynamic network and liquid crystalline properties of 6CC-2, 6SS-2 and 6SeSe-2 (a) normalized stress relaxation data at 25 °C (strain = 1%, parallel plate geometry) (b) order parameter as a function of strain for 6CC-2, 6SS-2 and 6SeSe-2 samples (c) 2D WAXS images of 6CC-2, 6SS-2 and 6SeSe-2 samples at 160% strain, double headed arrows indicate the direction of mesogen orientation.

Shear stress relaxation experiments on the LCEs were conducted at room temperature (25 °C) under 1% strain for 1 hour to assess how the dynamic bonds and liquid crystalline domain rotation contribute to the materials' dissipative behavior. After 1 hour, 6CC-2, 6SS-2, and 6SeSe-2 dissipated 44%, 50% and 67% of the applied stress respectively (Fig. 5a), highlighting an increase in stress relaxation with the decrease in bond strength. To quantify the alignment of mesogens as a function of strain, wide angle X-ray scattering (WAXS) experiments were performed with *in situ* tensile testing. The intensities of the WAXS images were plotted as a function of azimuthal angle from which order parameters (*S*) were calculated based on the Kratky method.^72^ The *S* values are expected to fall between 0 and 1, with an isotropic sample approaching a value of *S* = 0 and an aligned sample approaching a value of *S* = 1. To obtain the order parameter of polydomain samples (0% strain), WAXS was performed on each sample as loaded. The samples were then incrementally strained by 20% (strain rate = 1% per sec) up to a maximum of 160%, with WAXS performed after each strain increment. The order parameters for all three samples increased rapidly up to 80% strain, with an average Δ*S* = 0.34 from 0% to 80% strain. From 80% strain to 160% strain, there was only a minimal increase in order parameter, with an additional average Δ*S* = 0.07. This suggests that the alignment of mesogens mostly occurs during 0% to 80% strain. Above 80% strain, the order parameters are slightly lower for 6SeSe-2 compared to that of 6CC-2 and 6SS-2. As shown in Fig. 5b and c, at 160% strain, the order parameters for 6CC-2, 6SS-2, and 6SeSe-2 are *S* = 0.43, *S* = 0.44, *S* = 0.36 respectively, which might suggest the cleavage and exchange of diselenide bonds under high mechanical strain resulting in a decrease in overall alignment of mesogens.^70^

**Fig. 6:**
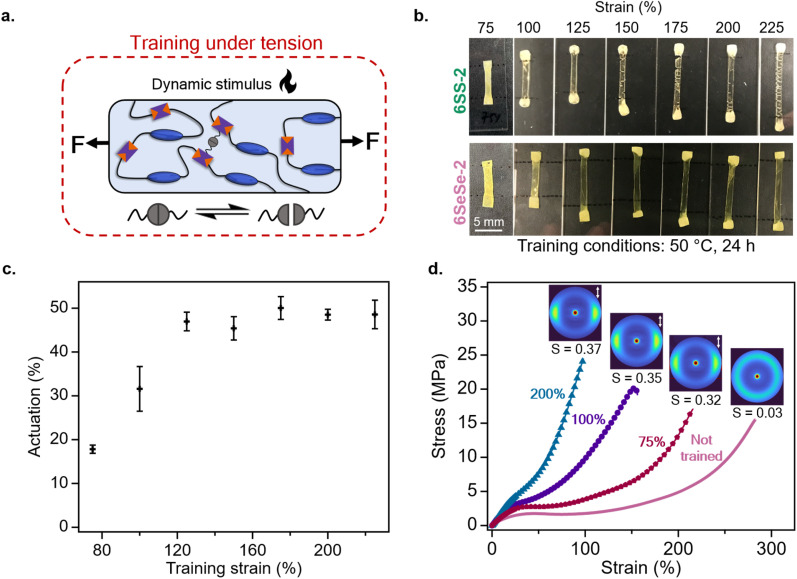
Trainability in dynamic LCEs under tension (a) schematic showing the training procedure, where heat is used to induce dynamic bond exchange (b) images showing 6SS-2 and 6SeSe-2 after training at 50 °C for 24 hours at different strains (c) actuation as a function of training strain in trained 6SeSe-2 samples (d) difference in mechanical properties and their corresponding WAXS images with order parameters as a function of training strain in trained 6SeSe-2 samples, double headed arrows indicate the direction of mesogen orientation.

With a better understanding of the properties of these LCEs, the next step was to explore their trainability as a function of mechanical strain. Fig. 6a shows the schematic of the training protocol, where the samples were strained to a controlled value beyond the soft elasticity, clamped between two glass slides and left at slightly elevated temperature (50 °C) for a specified time. When 6SS-2 films were held at strains ranging from 75% to 225% at 50 °C for 24 hours, multiple cracks formed in the materials, particularly at and above 150% strain, limiting the mechanical training of the 6SS-2 films up to 125% strain (Fig. 6b). At the training conditions used, the 6SS-2 samples that did not form cracks reverted back to polydomain states when they were heated above the *T*_NI_, thus they were not further characterized for actuation behavior or order parameter measurements. Similarly, 6CC-2 films formed cracks and failed when trained under similar conditions at and above 150% strain (Fig. S14). In contrast, the 6SeSe-2 films retained their integrity across all strain levels tested and could be successfully programmed into their monodomain states. This was evident from their reversible actuation behavior, which was determined based on their relaxed lengths at room temperature and their contracted lengths above the *T*_NI_ (Fig. 6c). Based on these results 6SeSe-2 was chosen to further study as the trainable system under different levels of mechanical strain. As shown in Fig. 6c, the actuation (%) gradually increased as the samples of 6SeSe-2 were trained from 75% to 125% strain, beyond which the actuation reached a plateau. At higher strains, the samples entered the strain-hardening regime (Fig. 3a), where minimal mesogen realignment occurs, resulting in negligible additional actuation. To investigate how training influences mesogen alignment, order parameters were calculated from the WAXS data. As shown in Fig. 6d, the not trained (polydomain) sample and the samples trained at 75%, 100%, and 200% strain exhibited order parameter values of *S* = 0.03, *S* = 0.32, *S* = 0.35, and *S* = 0.37 respectively. These samples were then uniaxially strained at 1% per sec until failure, where higher training strains resulted in a progressively stiffer mechanical response. Here, a range of actuation and mechanical responses was achieved in 6SeSe-2 by varying the degree of mesogen alignment through training the samples at different strain levels.

In addition, LCEs with lower crosslinking densities (*M*_c_ = 3.5 kg mol^−1^ and *M*_c_ = 5 kg mol^−1^) were also studied using the similar thermal/mechanical training protocol. Similar to 6CC-2 and 6SS-2, both 6CC-3.5 and 6SS-3.5 developed multiple cracks when trained at higher strains (Fig. S15). In contrast, 6SeSe-3.5 remained intact across all training strains. Likewise, 6CC-5 and 6SS-5 exhibited multiple cracks at higher training strains. Interestingly, 6SeSe-5 also developed cracks at higher strains, suggesting insufficient stress relaxation due to the lower concentration of diselenide bonds (Fig. S16). Uniaxially aligned LCEs have been observed to undergo stress relaxation at extended time scales primarily due to the non-mesogenic polymeric components.^73^ In this experimental set up the films are held under strain preventing stress relaxation by the polymer chains. However, for the more dynamic 6SeSe-2 the weaker bonds allow for stress relaxation to occur *via* bond exchange. For the 6CC-2 and 6SS-2 films on the other hand, the stronger bonds inhibit any stress relaxation *via* bond exchange, resulting in the samples breaking at a macroscopic scale to release the internal stress.

**Fig. 7:**
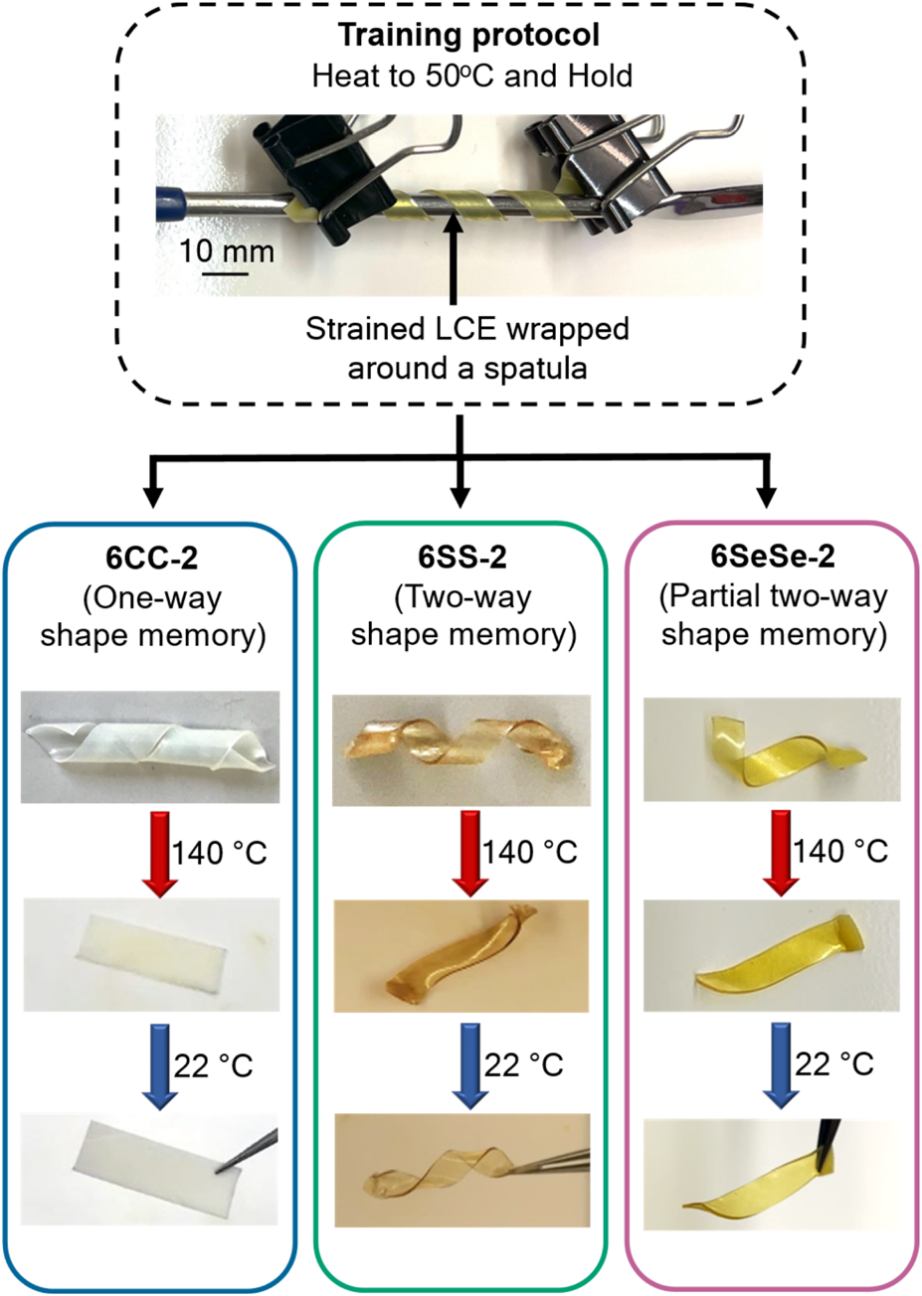
Functional actuators obtained by training dynamic LCEs. Rectangular strips of 6CC-2, 6SS-2, and 6SeSe-2 were strained to 125% strain and the strained films were wrapped around a metal spatula and left in the oven at 50 °C. 6SeSe-2 was trained for 7 hours, while 6SS-2 and 6CC-2 were trained for 48 hours each.

Next, mechanically strained films of 6CC-2, 6SS-2, and 6SeSe-2 were studied in the fabrication of shape memory actuators using a similar thermal/mechanical training protocol. First, to understand the stress relaxation behavior for each material type, shear stress relaxation experiments were performed on polydomain samples of 6CC-2, 6SS-2, and 6SeSe-2 at 50 °C under 1% strain for 1 hour. After 1 hour, 6CC-2 and 6SS-2 dissipated approximately 50% of the applied stress while 6SeSe-2 dissipated 70% stress (Fig. S17), consistent with faster exchange dynamics for 6SeSe-2 at 50 °C relative to 6CC-2 and 6SS-2. To obtain shape memory actuators *via* training, films of 6CC-2, 6SS-2, and 6SeSe-2 were strained to 125%, wrapped around a metal spatula and held at 50 °C (Fig. 7). 6SeSe-2 was trained for 7 hours, while 6CC-2 and 6SS-2 were trained for 48 hours each. After training, the samples were cooled to room temperature while still wrapped around the spatula. Upon removal from the spatula, the samples maintained their spiral shapes introduced during the training. When the 6CC-2 spiral was heated to 140 °C (above *T*_NI_), it exhibited one-way shape memory behaviour, irreversibly changing from its spiral shape back to a rectangular strip after a single heating and cooling cycle (Fig. 7 and Video S1). The lack of dynamic bond exchange in 6CC-2 at 50 °C, means the mesogen orientation could not be ‘locked in’ to the spiral pattern (Fig. S18). On the other hand, 6SS-2 and 6SeSe-2 were converted into two-way shape memory actuators, driven by the activation of disulfide and diselenide bonds, respectively (Fig. 7 and S19). When heated to 140 °C (above *T*_NI_), the actuators fabricated from 6SS-2 and 6SeSe-2 exhibited self-sustained “dancing” motion (Videos S2 and S3), which is triggered by the thermal gradient between the surfaces that are either in or out of contact with the hot plate. As the sample actuates, the centre of gravity constantly shifts from one end of the film to the other as it lifts and rapidly cools down, thus maintaining the thermo-mechanically induced self-sustained oscillation (Fig. S20). Upon cooling, the 6SS-2 actuator fully recovered its spiral shape, while the 6SeSe-2 actuator only partially recovered. This is likely a consequence of the increased creep from the dynamic exchange of the weaker diselenide bonds at higher temperature (Fig. S21 and Video S3).

**Fig. 8:**
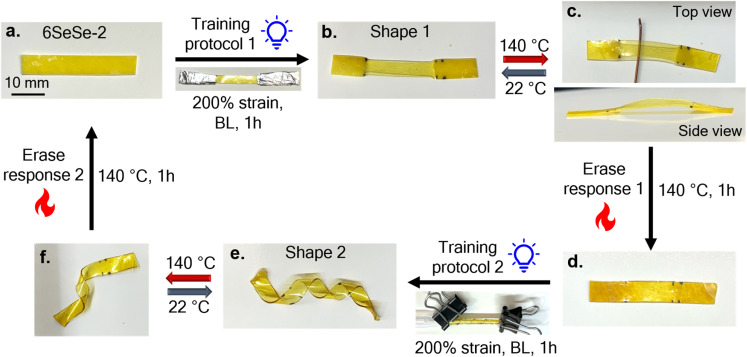
Images showing the ability to erase and retrain dynamic LCEs: (a) rectangular strip of 6SeSe-2, (b) Shape 1 obtained by using training protocol 1, where the sample is selectively trained in the middle by shielding the ends before exposure to blue light, (c) actuation response of Shape 1 showing bulging in the middle when heated above *T*_NI_, (d) memory of Shape 1 erased by heating the sample at 140 °C for 1 hour, (e) Shape 2 obtained by using training protocol 2, where the strained sample is wrapped around a glass pipette and exposed to blue light. (f) Actuation response of Shape 2 showing the spiral uncurling when heated above *T*_NI_.

Next, the retraining capabilities of 6SeSe-2 was investigated. Unlike in the previous experiments (Fig. 6 and 7), which used thermal activation at 50 °C, blue light was employed here to spatially control actuation, leveraging the visible-light responsiveness of the diselenide bonds.^55^ To evaluate retrainability, a rectangular strip of 6SeSe-2 (Fig. 8a) was selectively aligned in the centre by stretching the sample to 200% strain, shielding the ends with aluminium foil, and irradiating with blue light (7.5 mW cm^−2^) for 1 hour. This training procedure produced a programmed configuration referred to as Shape 1 (Fig. 8b), in which the mesogens were aligned in the trained center, while remaining polydomain in the untrained ends (Fig. S22). When Shape 1 was placed on a hot plate at 140 °C (above the *T*_NI_), differences in the degree of mesogen alignment across the sample caused it to bulge in the center (Fig. 8c and S23). This bulging behavior can result from a temperature gradient formed between the lower and upper sides of the aligned center. When the sample is placed on the hotplate, the lower surface gets heated above the *T*_NI_ first resulting in a contraction of the lower side of the film before the upper side, which leads to the formation of arch. When a replica of Shape 1 was placed inside an oven at 140 °C, the sample remained mostly flat, further supporting that the bulging behavior arises from the temperature gradient across the sample (Fig. S24). Similar actuation behavior has been reported in the literature with the explanation corroborated by finite element analysis.^74^ Upon cooling, the sample reverted to Shape 1. To erase the programmed alignment, the sample was heated at 140 °C for 1 hour (Fig. 8d). A new shape was then introduced by retraining the same sample using a different training protocol. The film was re-strained to 200%, wrapped around a glass pipette, and irradiated again with blue light (7.5 mW cm^−2^) for 1 hour. This process resulted in a spiral actuator, referred to as Shape 2 (Fig. 8e). When Shape 2 was heated above the *T*_NI_, the actuator rapidly attempted to uncurl (Video S4), and upon cooling, it returned to its spiral configuration. The full recovery of Shape 2 in Video S4, compared to the partial recovery of the 6SeSe-2 actuator in Video S3, is attributed to the difference in the duration each sample was exposed to heat. The 6SeSe-2 actuator in Video S3 remained on the hot plate for approximately 190 seconds, allowing for more creep, whereas Shape 2 in Video S4 was heated for only about 45 seconds. The fabrication of two distinct actuators from a single sample demonstrated the capability to train, erase, and retrain 6SeSe-2.

## Conclusions

In this work, the trainability of dynamic LCEs was demonstrated by studying a series of aza-Michael based LCEs, where the strength of the dynamic bonds was tuned by incorporating diamine crosslinkers containing either no dynamic bonds, disulfide bonds, or diselenide bonds. While variations in dynamic bond strength had minimal influence on the thermal transitions and the viscoelastic response of the LCEs, the diselenide-containing system exhibited markedly higher toughness relative to the other two systems. In addition, by training diselenide LCEs under different levels of mechanical strain, a range of actuation as well as mechanical properties were achieved. Furthermore, both disulfide and diselenide LCEs were trained to fabricate spiral actuators that showed self-sustained ‘dancing’ motion when heated above their *T*_NI_'s. Notably, two distinct actuator shapes were obtained from a single diselenide LCE sample, demonstrating retrainability through spatially selective training enabled by blue light. These findings highlight the potential of dynamic LCEs as versatile trainable materials, with tunable training strategies enabling a wide range of functional properties.

## Author contributions

E. G. and S. J. R. conceptualized the idea. E. G. performed the experiments and analysed the results. I. S. A and L. B. de A. e S synthesized LCEs. E. G., C. A. L and S. J. R. wrote and edited the manuscript. C. A. L and S. J. R. supervised the project. S. J. R. provided financial support for the project.

## Conflicts of interest

There are no conflicts to declare.

## Supplementary Material

SC-017-D5SC07491F-s001

SC-017-D5SC07491F-s002

SC-017-D5SC07491F-s003

SC-017-D5SC07491F-s004

SC-017-D5SC07491F-s005

## Data Availability

The data supporting this study, including the synthesis and characterization of crosslinker and LCEs, training studies, and actuation measurements, have been included within the article and supplementary information (SI). Supplementary information: experimental details, materials and methods, and additional characterization of LCEs including TGA, DSC thermograms, IR spectroscopy measurements, tensile curves with highlighted soft elastic regions, stress relaxation and creep experiments, WAXS images, NMR spectra and videos showing the shape memory behavior of the spiral LCE films. See DOI: https://doi.org/10.1039/d5sc07491f.
