# Supplementary material for: Impact of dynamic bond strength on the training of liquid crystal elastomers
Source: Chem Sci. 2025 Dec 4;17(4):2263–72. doi: 10.1039/d5sc07491f (PMC12679475; doi:10.1039/d5sc07491f)
Supplement: SC-017-D5SC07491F-s001 [file SC-017-D5SC07491F-s001.pdf]

Supplementary Information (SI) for Chemical Science.
This journal is © The Royal Society of Chemistry 2025

Electronic Supplementary Information

Impact of dynamic bond strength on the training of liquid crystal elastomers

Elina Ghimire[a], Ingrid S. Appen[a], Charlie A. Lindberg[a], Leticia Blagitz de Abreu e Silva[b], Stuart J. Rowan[a,c,d,*]

[a]*Pritzker School of Molecular Engineering, University of Chicago, Chicago, Illinois 60637, USA*
[b]*University of Chicago Laboratory Schools, Chicago, Illinois, 60637, USA*
[c]*Department of Chemistry, University of Chicago, Chicago, Illinois 60637, USA*
[d]*Center for Molecular Engineering, Argonne National Laboratory, 9700 S. Cass Ave., Lemont, IL 60434, USA*

* *to whom correspondence should be addressed:* stuartrowan@uchicago.edu

Table of Contents

EXPERIMENTAL DETAILS

Materials S3

Instrumentation S3-S4

Synthesis S4

Preparation of LCE films S4

Equation used S5

CHARACTERIZATION

Table S1 Chemical compositions of different LCEs studied S6

Figure S1 Stacked TGA data S6

Figure S2 IR Spectroscopy measurements of LCE films S7

Table S2. Gel fraction and thermal transitions of LCEs S7

Figure S3. DSC thermograms of 6CC materials S8

Figure S4. DSC thermograms of 6SS materials S8

Figure S5. DSC thermograms of 6SeSe materials S8

Fig S6. Frequency sweep measurements for 6CC-2 S9

Fig S7. Frequency sweep measurements for 6SS-2 S9

Fig S8. Frequency sweep measurements for 6SeSe-2 S9

Fig S9. (a) Tensile curve for 6SeSe-2 with three tangent lines (b) Tensile curves highlighting regions of soft elastic plateau S10

Fig S10. Tensile curves for 6CC-2, 6CC-3.5, 6CC-5 S10

Fig S11. Tensile curves for 6SS-2, 6SS-3.5, 6SS-5 S10

Fig S12. Tensile curves for 6SeSe-2, 6SeSe-3.5, 6SeSe-5 S11

Figure S13. Images of 6CC-2, 6SS-2, and 6SeSe-2 samples in polydomain state and after stretching S11

Fig S14. Images showing 6CC-2 after training at 50 °C for 24 hours S11

Fig S15. Images showing 6CC-3.5, 6SS-3.5, and 6SeSe-3.5 after training at 50 °C for 24 hours S12

Fig S16. Images showing 6CC-5, 6SS-5, and 6SeSe-5 after training at 50 °C for 24 hours S12

Fig S17. Normalized stress relaxation data at 50 °C S13

Fig S18. Schematic of one-way shape memory behavior observed in 6CC-2 S13

Fig S19. Schematic of one-way shape memory behavior observed in 6SS-2 and 6SeSe-2 S13

Fig S20. Thermo-mechano feedback loop in 6SS-2 LCE S14

Fig S21. Creep measurements of 6SS-2 and 6SeSe-2 at 120 °C with 1 MPa load S14

Fig S22. 2D WAXS images and order parameters of Shape 1 shown in Fig 8b S15

Fig S23. Schematic showing mesogen orientation and actuation behavior in different regions of Shape 1 shown in Fig 8b S15

Fig S24. A replica of 6SeSe-2 Shape 1 that exhibits bulging behavior on a hot plate, however remains mostly flat inside an oven at the same temperature S16

NMR spectra S17

References S18

Supporting videos S18

EXPERIMENTAL DETAILS

Materials

2-Methyl-1,4-phenylene bis(4-((6-(acryloyloxy)hexyl)oxy)benzoate) (RM 82) (LC monomer) was purchased from Daken Chemical Limited. 4-Nitrophenol was purchased from Fluka. All other reagents were purchased from Sigma-Aldrich and were used as received unless otherwise noted. All solvents were purchased from Fisher Scientific and were used as received unless otherwise noted.

Instrumentation

Nuclear Magnetic Resonance (NMR)- All NMR spectra were collected on Bruker Avance III HD nanobay 400 MHz spectrometer.

Thermogravimetric Analysis (TGA)- TA Instruments Discovery Thermogravimetric Analyzer was used to perform TGA experiments, where the samples were heated from 25 °C to 600 °C at the rate of 10 °C/min.

Differential Scanning Calorimetry (DSC)- TA Instruments Discovery 2500 differential scanning calorimeter was used to perform DSC measurements. The samples were sealed in aluminum hermetic pans (TA Instruments), and DSC was conducted using the following thermal profile: heating cycle (22 °C/200 °C), cooling cycle (200 °C/-90 °C), heating cycle (-90 °C/200 °C), and cooling cycle (200 °C/-90 °C). All heating and cooling ramps were performed at a rate of 10 °C/min. Data from the second heating cycle are presented in this paper.

Tensile Test- Zwick-Roell zwickiLine Z0.5 was sued to collect all tensile test data. The samples were prepared by cutting rectangular strips (~ 5mm x 0.5mm x 20mm) from the melt pressed films. The samples were uniaxially stretched at the rate of 1 %/sec at room temperature.

<u>Creep measurement-</u> The samples were mounted on the tensile grips, maintained the desirable environmental condition and stretched at the rate of 200 mm/min until the force reached 500 kPa. The force was held at 500 kPa

for 1 hour while monitoring the strain. After 1 hour, the force was released, and the strain was recorded for another 1 hour. The measurements were performed either at ambient condition, at 50 °C or 120 °C in the Zwick oven or by shining blue light in front of the sample using table lamp.

Shear Rheology- TA Instruments ARES-G2 shear rheometer was used to collect stress relaxation and dynamic temperature ramp measurements.

<u>Stress relaxation-</u> 8 mm discs were punched from the as-pressed films and mounted between parallel plate geometries. The samples were heated to 160 °C and annealed for 5 minutes, followed by cooling to 25 °C. A 1% strain, within the linear viscoelastic regime, was then applied and held for 1 hour while monitoring the stress response. Throughout the measurement, a constant axial compressive force of 4 N was applied to ensure consistent contact between the sample and the plates.

<u>Dynamic temperature ramp-</u> 8 mm discs, punched from the as-pressed films, were mounted between parallel plate geometries. The samples were then heated to 160 °C, held for 5 minutes, and cooled to -20 °C. Subsequently, the temperature was ramped to 180 °C at a rate of 3 °C/min under a constant frequency of 1 Hz and a strain of 0.01%.

Wide Angle X-ray Scattering (WAXS)- All WAXS data was collected using a SAXSLAB GANESHA 300XL using a Cu Kα source (λ = 0.154 nm) at a power of 40 mA and a voltage of 40 kV. Film samples were mounted on the holder using Kapton tape and shot directly for 3 mins at q = 0.05 – 2.5 $Å^{-1}$.

<u>WAXS Data Processing-</u> For each sample, four WAXS patterns were acquired by shifting the detector to capture the complete 2D scattering profile. The images were aligned, averaged using a polar transformation, and then inverse transformed to reconstruct the final scattering pattern. A distinct peak at q ≈ 1.5 $Å^{-1}$ was observed in the 1D plot, corresponding to an average mesogen spacing of d ≈ 4.2 Å. To determine the order parameter, the 2D data were azimuthally integrated over the q-range of 0.97–1.90 $Å^{-1}$ to generate a 1D plot of intensity versus angle. Order parameters were then calculated using the Kratky method as previously described.[1]

Infrared Spectroscopy (IR)- Shimadzu IRTracer-100 FT-IR with ATR diamond was used to collect all IR spectra.

<u>Synthesis</u>

Diselenide diphthalimide- First, Na_2Se_2 was synthesized using a previously reported procedure.[2] 3g Se and 1 g of $NaBH_4$ were added in a 250 ml three-neck round bottom flask, which was placed in an ice bath. A waterless condenser was attached to one of the necks, and a nitrogen line was connected to one of the other two necks. Under nitrogen flow, 100 ml of ethanol was added to the flask dropwise. Extra precaution was maintained during the addition of ethanol as the reaction is very exothermic. The reaction was refluxed at 80 °C for 1.5 h resulting in a dark red liquid. Without isolating Na_2Se_2, the reaction was continued to the next step where 6.03g of *N*-(2-Bromoethyl)phthalimide was added in the flask and allowed to reaction for 18 h. Greenish yellow solid was precipitated after the end of the reaction, which was filtered, then washed 3-4 times with ethanol. The residue was then dissolved in DCM and filtered again. The filtrate was collected and the solvent removed using a rotary evaporator before being dried under vacuum to obtain yellow solid as the product (5.62g, 54.6% yield).

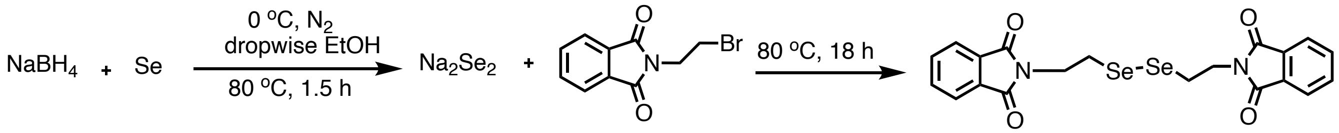

Diselenide diamine- 2g of diselenide diphthalimide was dissolved in 100 ml of anhydrous THF. 11.75 ml of hydrazine hydrate (50-60% concentration) was added to the solution dropwise and the mixture was allowed to react for 18 h. A white byproduct crashed out, which was filtered out. The yellow filtrate was collected and the solvent removed using a rotary evaporator before being and dried under vacuum to obtain the product as a dark yellow oil (0.8g, 40.7% yield).

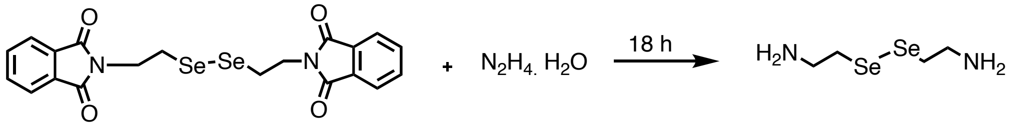

<u>Preparation of 6CC, 6SS and 6SeSe</u>

A 1:1 molar ratio of acrylates to amines was used to maintain stoichiometric balance, with all acrylates provided by the liquid crystalline monomer RM 82. The amines were split between hexamethylenediamine (as the crosslinker) and hexylamine (as the chain extender). Component masses (Table S1) were combined in a vial with 6 mL of toluene. *p*-nitrophenol catalyst was added to the mixture, which was sonicated to aid dissolution, then purged with nitrogen gas for 15 minutes after which the samples were cured at 60 °C. After 16 hours, LCE gels formed and were subsequently washed overnight in acetone using a Soxhlet apparatus. The washed gels were dried at 60 °C overnight. Final films were produced by melt-pressing the gels under 4 tons of pressure: **6CC** and **6SS** at 160 °C, and **6SeSe** at 130 °C, each for 1 hour. The resulting films measured approximately 50 mm × 0.5 mm × 50 mm.

<u>Equation Used</u>

Calculation of actuation %

% actuation = $\frac{L_R - L_C}{L_R} * 100$ (1) Where, L_R is the length of the programmed LCE film at room temperature

L_C is the length of the programmed LCE film above the T_{NI}

CHARACTERIZATION

Table S1. Chemical compositions of different LCEs studied

Molecular weight between crosslinks (M_c) (g/mol)	LCE sample	LC monomer (RM 82) (g)	Chain extender (HA) (g)	Crosslinker (CC diamine, SS diamine, SeSe diamine) (g)	Catalyst (*p*-nitrophenol) (g)
2000	**6CC-2**	4.036	0.159	0.256	0.083
	**6SS-2**	4.036	0.159	0.336	0.083
	**6SeSe-2**	4.036	0.159	0.543	0.083
3500	**6CC-3.5**	4.036	0.346	0.149	0.083
	**6SS-3.5**	4.036	0.346	0.196	0.083
	**6SeSe-3.5**	4.036	0.346	0.317	0.083
5000	**6CC-5**	4.036	0.423	0.105	0.083
	**6SS-5**	4.036	0.423	0.138	0.083
	**6SeSe-5**	4.036	0.423	0.224	0.083

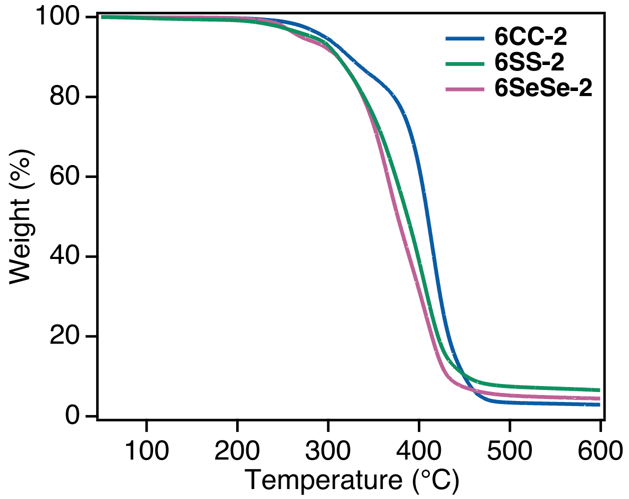

Fig S1. TGA curves for **6CC-2**, **6SS-2**, and **6SeSe-2**

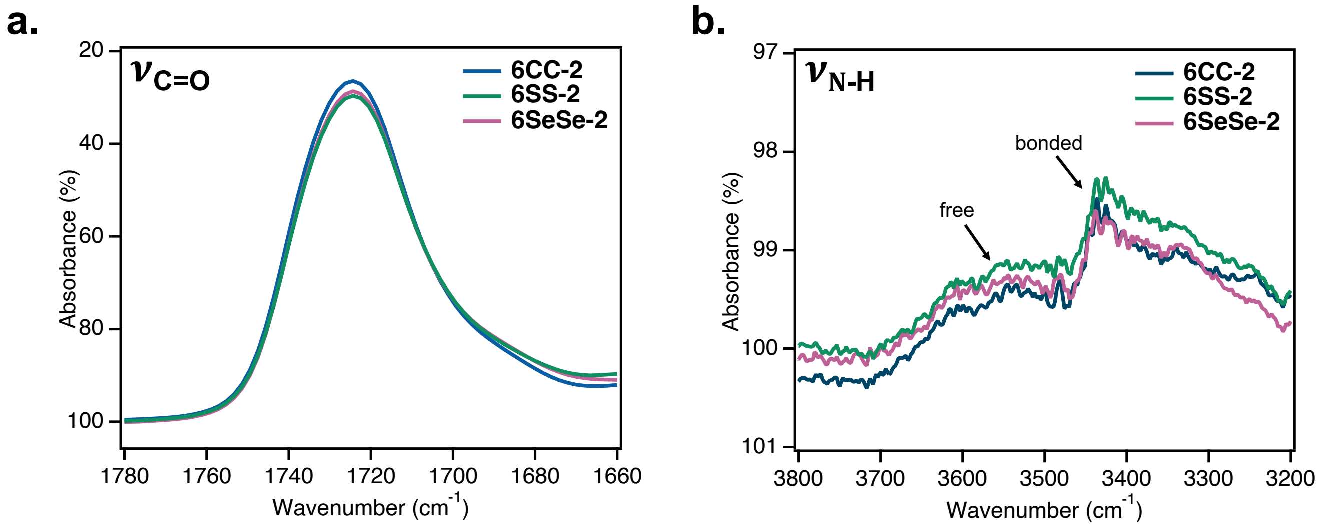

Fig S2. FTIR spectra for **6CC-2**, **6SS-2**, and **6SeSe-2** showing insignificant H-bonding in all samples (a) C=O stretch (b) N-H stretch

Table S2. Gel fraction and thermal transitions of LCEs

LCE sample	Gel fraction (%)	T_g (°C) (DSC, 2nd heat)	T_{NI} (°C) (DSC, 2nd heat)
**6CC-2**	92.47 ± 2.99	-1.52 ± 0.27	97.37 ± 3.92
**6CC-3.5**	91.02	-9.84	87.73
**6CC-5**	89.94	-15.12	86.20
**6SS-2**	79.86 ± 8.54	0.11 ± 0.49	100.63 ± 2.38
**6SS-3.5**	74.78	-9.25	89.81
**6SS-5**	81.76	-14.31	84.23
**6SeSe-2**	76.90 ± 3.11	1.32 ± 0.39	110.42 ± 0.49
**6SeSe-3.5**	79.09	-9.44	94.19
**6SeSe-5**	86.01	-14.38	88.94

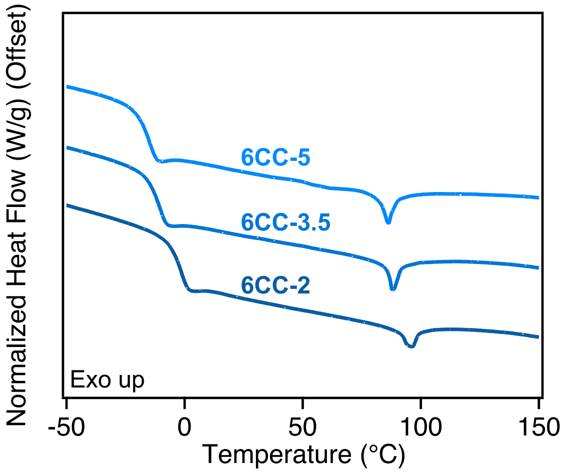

Fig S3. DSC thermograms for **6CC-2**, **6CC-3.5**, **6CC-5**

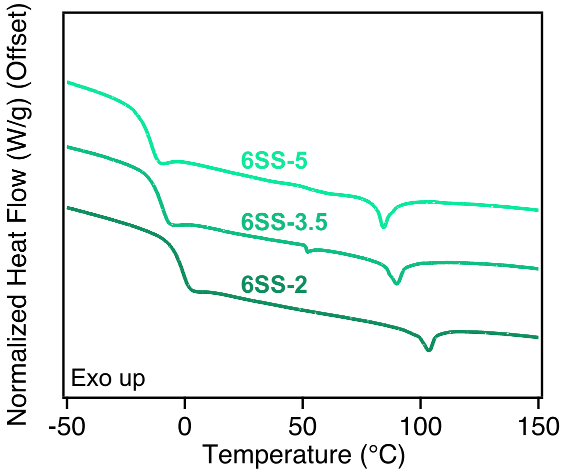

Fig S4. DSC thermograms for **6SS-2**, **6SS-3.5**, **6SS-5**

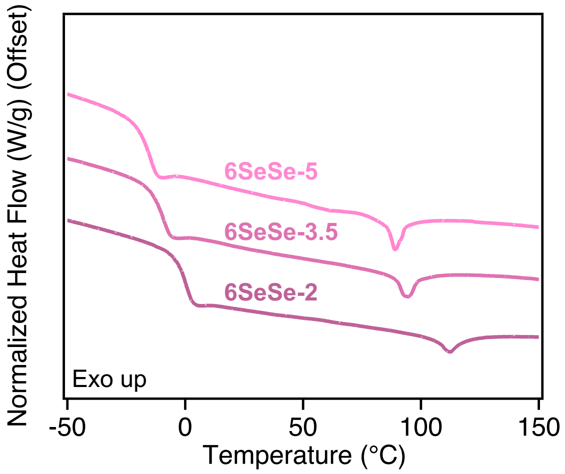

Fig S5. DSC thermograms for **6SeSe-2**, **6SeSe-3.5**, **6SeSe-5**

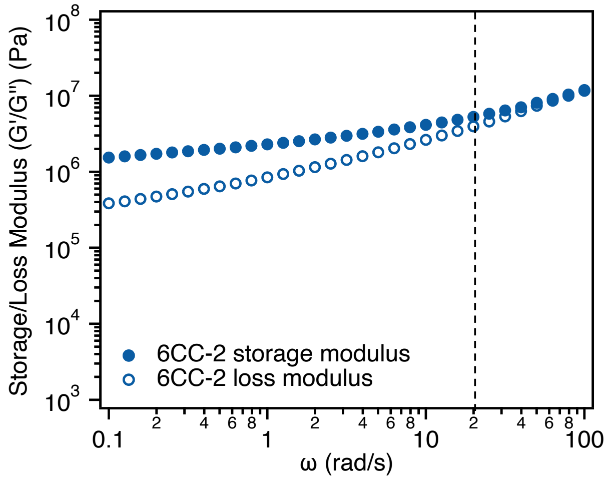

Fig S6. Frequency sweep measurements for **6CC-2**

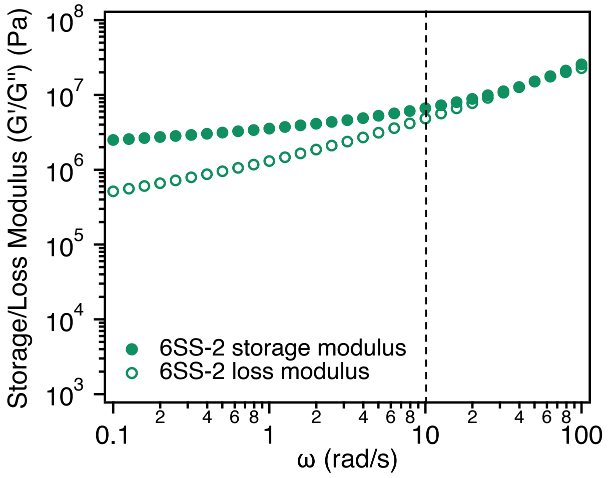

Fig S7. Frequency sweep measurements for **6SS-2**

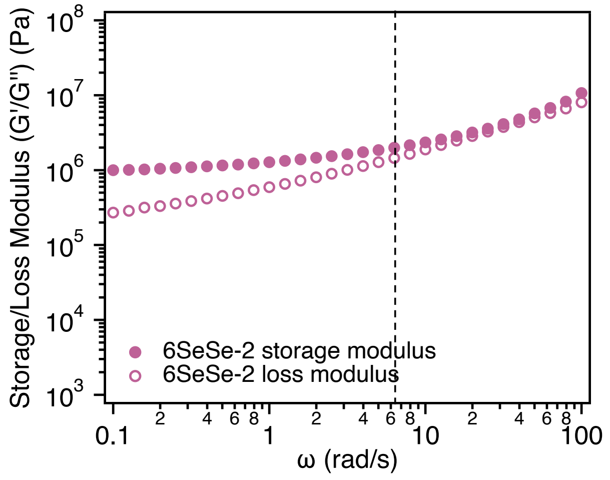

Fig S8. Frequency sweep measurements for **6SeSe-2**

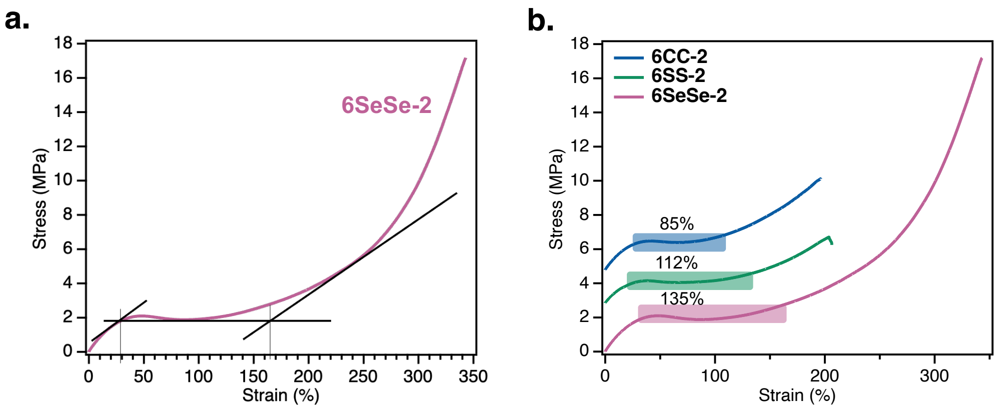

Fig S9. (a) Tensile curve for 6SeSe-2 with three tangent lines, which are used to determine the range of soft elastic plateau (b) Tensile curves highlighting regions of soft elastic plateau for **6CC-2**, **6SS-2**, and **6SeSe-2**

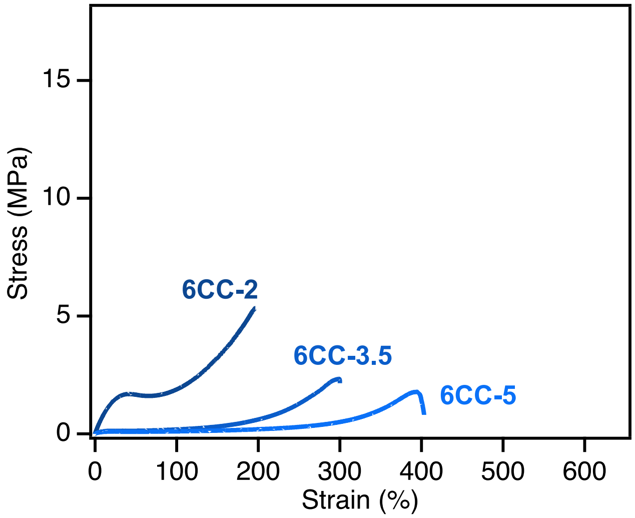

Fig S10. Tensile curves for **6CC-2**, **6CC-3.5**, **6CC-5**

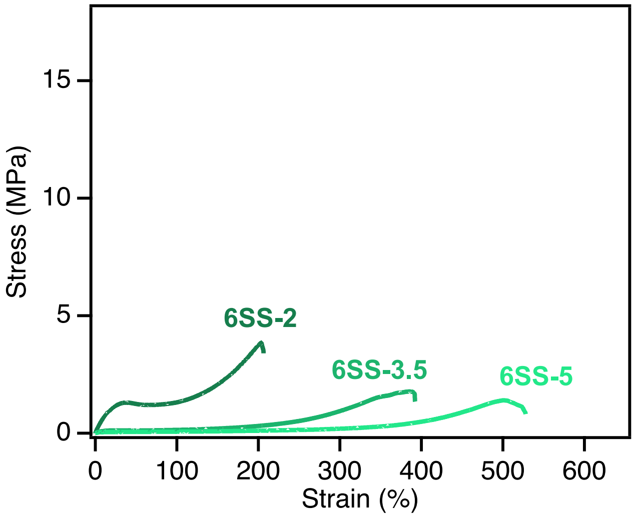

Fig S11. Tensile curves for **6SS-2**, **6SS-3.5**, **6SS-5**

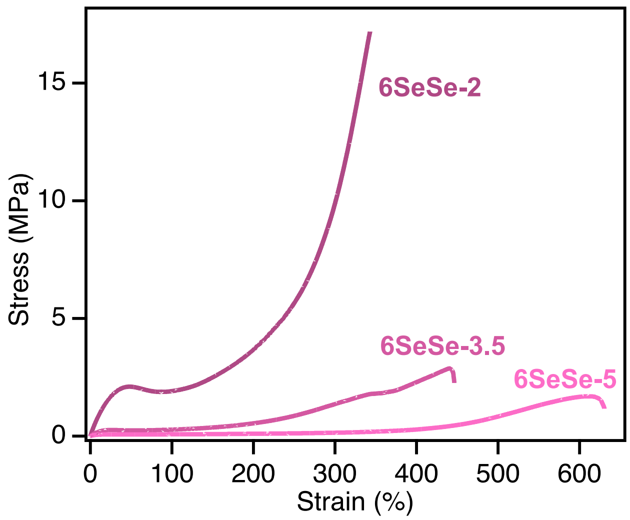

Fig S12. Tensile curves for **6SeSe-2**, **6SeSe-3.5**, **6SeSe-5**

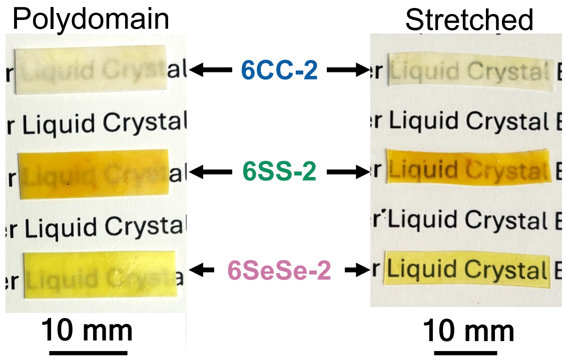

Figure S13. Images of **6CC-2**, **6SS-2**, and **6SeSe-2** samples in polydomain state and after stretching

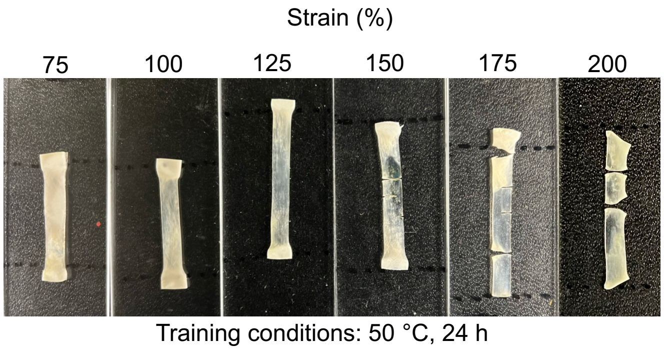

Fig S14. Images showing **6CC-2** after training at 50 °C for 24 hours at different strains

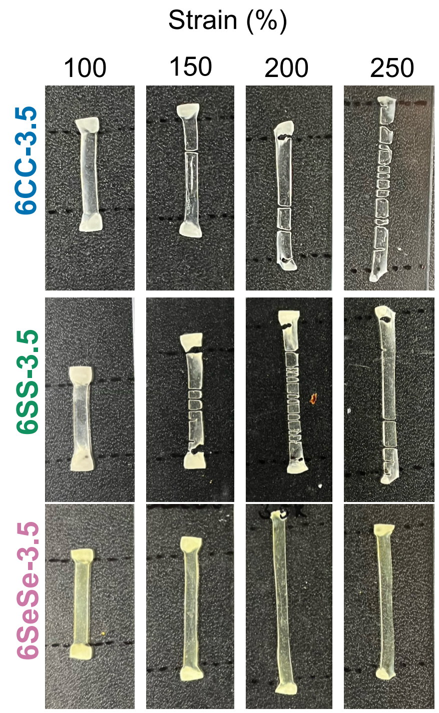

Fig S15. Images showing **6CC-3.5**, **6SS-3.5**, **and 6SeSe-3.5** after training at 50 °C for 24 hours at different strains

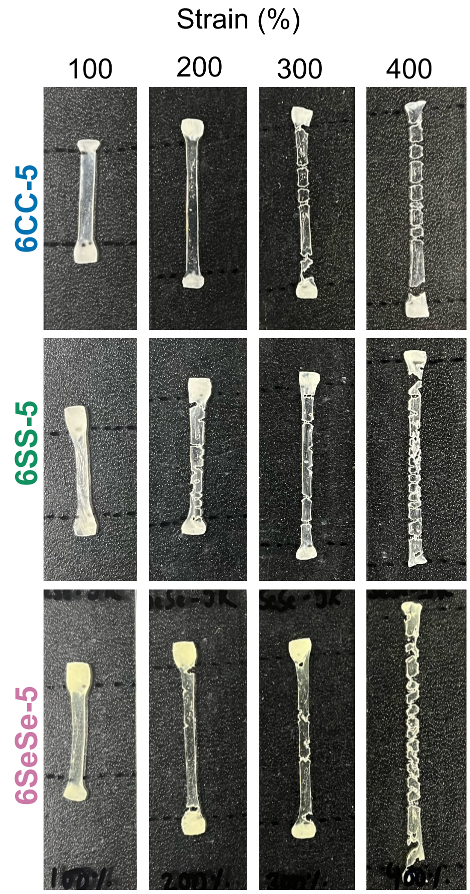

Fig S16. Images showing **6CC-5**, **6SS-5**, **and 6SeSe-5** after training at 50 °C for 24 hours at different strains

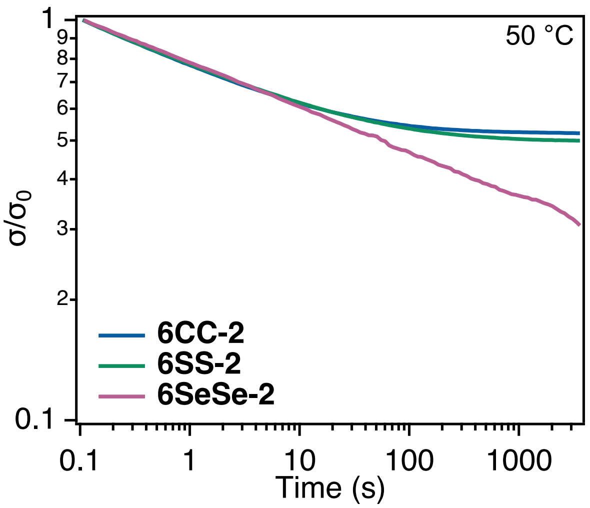

Fig S17. Normalized stress relaxation data at 50 °C (strain = 1%, frequency = 1 Hz, parallel plate geometry) for **6CC-2**, **6SS-2**, **6SeSe-2**

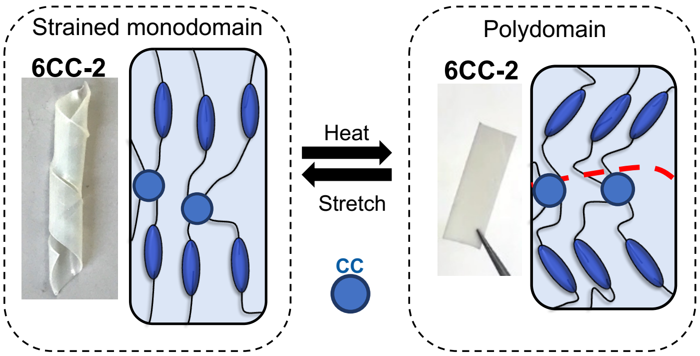

Fig S18. Schematic of one-way shape memory behavior observed in **6CC-2**

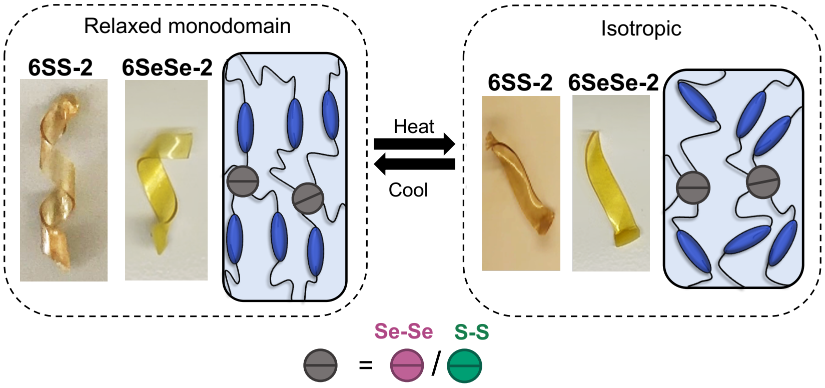

Fig S19. Schematic of one-way shape memory behavior observed in **6SS-2** and **6SeSe-2**

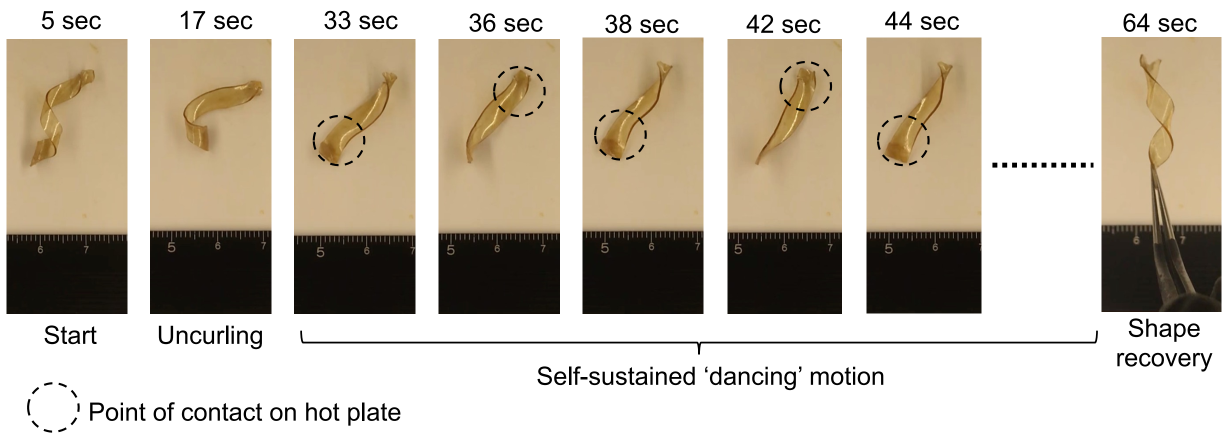

Fig S20. Thermo-mechano feedback loop in **6SS-2** LCE

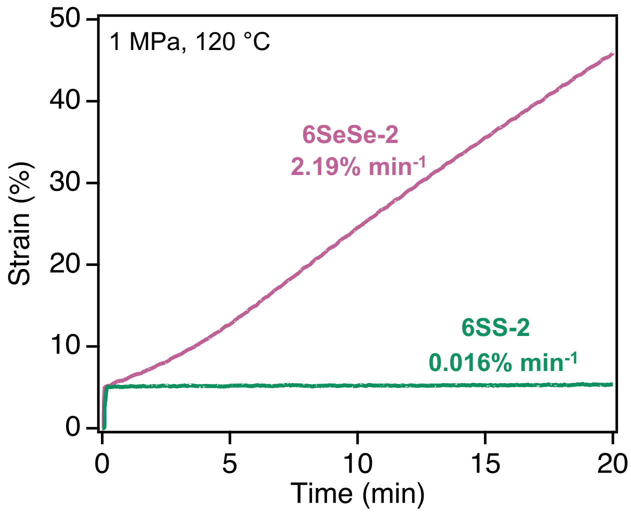

Fig S21. Creep measurements of **6SS-2** and **6SeSe-2** at 120 °C with 1 MPa load

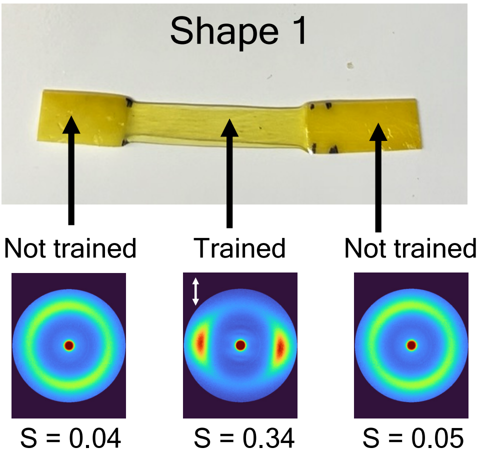

Fig S22. 2D WAXS images and order parameters of Shape 1 shown in Fig 8b, double headed arrow indicates the direction of mesogen orientation

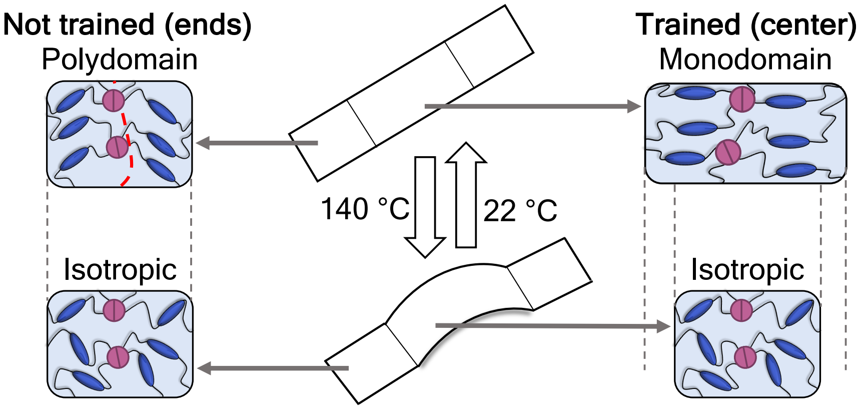

Fig S23. Schematic showing mesogen orientation and actuation behavior in different regions of Shape 1 shown in Fig 8b

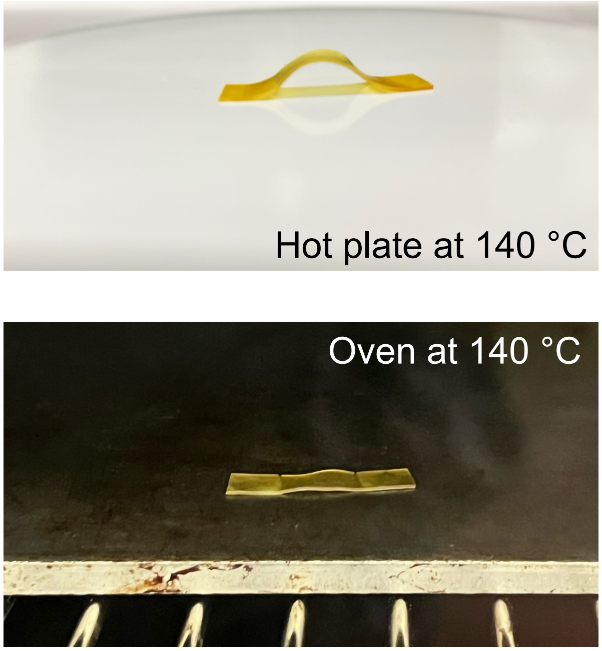

Fig S24. A replica of 6SeSe-2 Shape 1 that exhibits bulging behavior at 140 °C on a hot plate, however remains mostly flat when placed inside an oven at the same temperature

NMR spectra

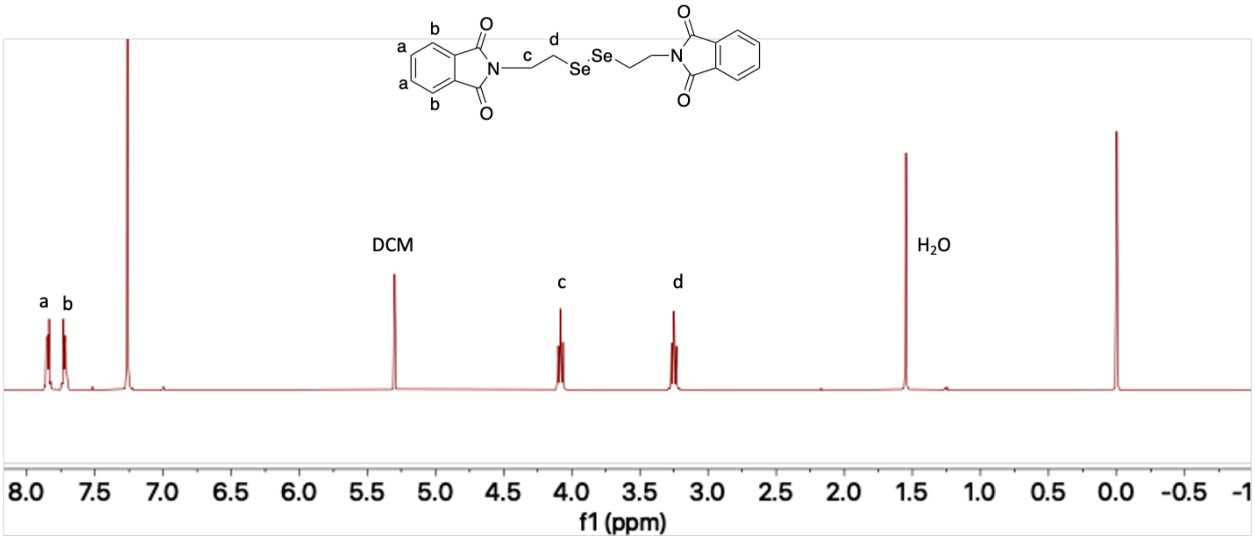
a
b
O
N
c
d
Se
Se
DCM
H2O
8.0 7.5 7.0 6.5 6.0 5.5 5.0 4.5 4.0 3.5 3.0 2.5 2.0 1.5 1.0 0.5 0.0 -0.5
f1 (ppm)

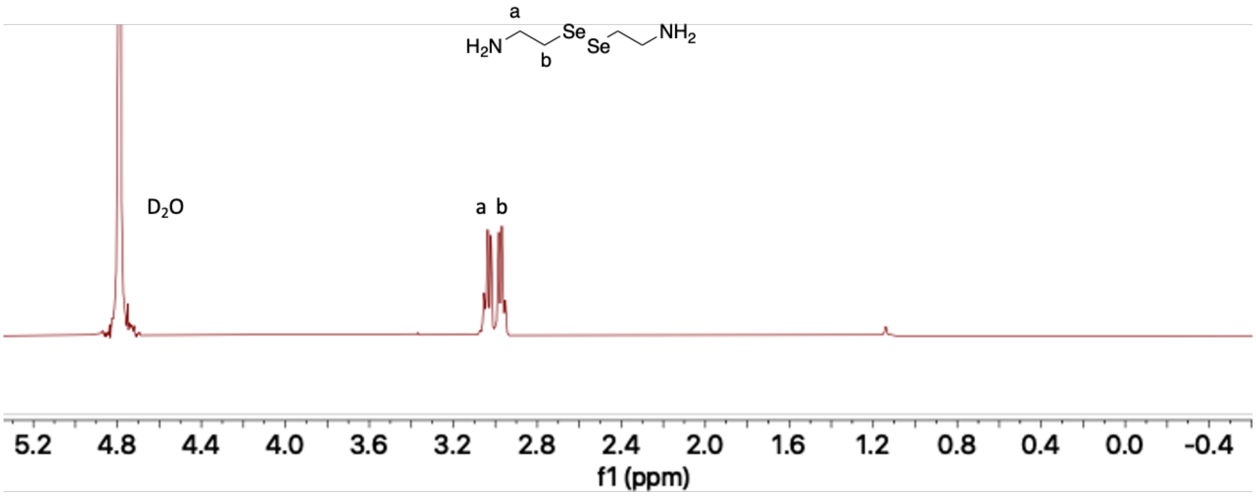
a
H2N
b
Se
Se
NH2
D2O
a b
5.2 4.8 4.4 4.0 3.6 3.2 2.8 2.4 2.0 1.6 1.2 0.8 0.4 0.0 -0.4
f1 (ppm)

References

(1) Sims, M. T.; Abbott, L. C.; Richardson, R. M.; Goodby, J. W.; Moore, J. N. Considerations in the Determination of Orientational Order Parameters from X-Ray Scattering Experiments. *Liq Cryst* **2019**, *46* (1), 11–24.

(2) Choi, D.; Lee, S.; Lee, J.; Cho, K. S.; Kim, S. W. Disodium Diselenide in Colloidal Nanocrystals: Acting as an Anion Exchange Precursor, a Metal Selenide Precursor, and a Chalcogenide Ligand. *Chemical Communications* **2015**, *51* (5), 899–902.

Supporting videos:

Video S1 Irreversible actuation of spiral shaped **6CC-2** actuator

The video shows the response of a spiral shaped actuator obtained from **6CC-2** when placed on a hot plate at 140 °C. Upon contact with the hot plate, the spiral shape uncurls into a rectangular film (0.04 – 0.10 s), which is then picked up with a spatula (0.11 – 0.14 s). After cooling, the sample remains as a rectangular film and loses its spiral shape. Still images of the movie are represented in Fig 7 of the main text. Video is played at 2× speed.

Video S2 Reversible actuation of spiral shaped **6SS-2** actuator

The video shows the response of a spiral shaped actuator obtained from **6SS-2** when placed on a hot plate at 140 °C. Immediately upon contact, the spiral begins to uncurl (0.01 – 0.08 s). This is followed by self-sustained actuation, where the two ends of the film alternately lift off and return to the surface of the hot plate (0.08 – 0.15 s). Finally, the actuator is lifted with a spatula, and as it cools, it recovers its original spiral shape (0.15 – 0.17 s). Still images of the movie are represented in Fig 7 of the main text. Video is played at 4× speed.

Video S3 Reversible actuation of spiral shaped **6SeSe-2** actuator

The video shows the response of a spiral shaped actuator obtained from **6SeSe-2** when placed on a hot plate at 140 °C. Immediately upon contact, the spiral begins to uncurl (0.01 – 0.06 s). During this process, the sample completely flips once and lands on a new location on the hot plate (0.01 – 0.07 s). This is followed by self-sustained actuation, where the two ends of the film alternately lift off and return to the surface of the hot plate (0.07 – 0.23 s). Finally, the actuator is lifted with a spatula, and as it cools, it partially recovers its original spiral shape (0.23 – 0.24 s). Still images of the movie are represented in Fig 7 of the main text. Video is played at 8× speed.

Video S4 Reversible actuation of **6SeSe-2** Shape 2 shown in Fig 8e

The video shows the response of Shape 2 (Fig 8e) when placed on a hot plate at 140 °C. Immediately upon contact, the two ends of the spiral uncurl (0.01 – 0.10 s), while the central region remains constrained. The sample is then lifted with a spatula, and as it cools, it recovers its original spiral shape (0.10 – 0.13 s). Still images of the movie are represented in Fig 8 of the main text. Video is played at 4× speed.
